# Barriers and Facilitators of Pain Management in Persons With Dementia in Long-Term Care: A Scoping Review

**DOI:** 10.1093/geroni/igab046.629

**Published:** 2021-12-17

**Authors:** Yo-Jen Liao, Ying-Ling Jao, Diane Berish, Lisa Kitko

**Affiliations:** 1 Penn State University, University Park, Pennsylvania, United States; 2 Pennsylvania State University, University Park, Pennsylvania, United States; 3 Pennsylvania State University, Pennsylvania State University, Pennsylvania, United States

## Abstract

Approximately 50% of individuals with dementia regularly experience moderate to severe pain, which is largely undermanaged. Several studies have explored the barriers and facilitators of pain management for persons with dementia; yet the evidence has not been systematically reviewed. This review aimed to synthesize current evidence on the barriers and facilitators of pain management in persons with dementia in long-term care. A PRISMA guided literature search was conducted in PubMed, CINAHL, and PsycINFO. Titles, abstracts, and full texts were screened. Included articles were original research examining the barriers or facilitators of pain assessment and treatment in individuals with dementia in long-term care. Quality assessment was conducted using the Risk of Bias tool and Johns Hopkins Level of Evidence. Ten studies were identified, including four quantitative studies, five qualitative studies, and one with both quantitative and qualitative research. Barriers of pain management identified include residents’ ability to self-report pain, pain medication side effects, need discrepancy among residents and their families, reluctance in administering analgesics, lack of pain assessment tools, lack of guidance in providing nonpharmacological interventions, and lack of clinical guidelines. Facilitators of pain management include clinicians with caring and enthusiastic characteristics, clinicians’ knowledge of residents, positive relationships among clinicians, good communication skills, using validated pain assessment tools, understanding pain indicators, clinical experience, and need-driven continuing education. These results can guide clinical practice in long-term care. Interventions should be developed to target these barriers and facilitators and improve pain management in persons with dementia.

